# Testis-enriched ferlin, FER1L5, is required for Ca^2+^-activated acrosome reaction and male fertility

**DOI:** 10.1126/sciadv.ade7607

**Published:** 2023-01-25

**Authors:** Akane Morohoshi, Haruhiko Miyata, Keizo Tokuhiro, Rie Iida-Norita, Taichi Noda, Yoshitaka Fujihara, Masahito Ikawa

**Affiliations:** ^1^ Research Institute for Microbial Diseases, Osaka University, Suita, Osaka 5650871 Japan.; ^2^ Graduate School of Medicine, Osaka University, Suita, Osaka 5650871 Japan.; ^3^ Institute of Biomedical Science, Kansai Medical University, Hirakata, Osaka 5731191 Japan.; ^4^ Institute of Resource Development and Analysis, Kumamoto University, Kumamoto, Kumamoto 8600811 Japan.; ^5^ Priority Organization for Innovation and Excellence, Kumamoto University, Kumamoto, Kumamoto 8608555 Japan.; ^6^ Department of Bioscience and Genetics, National Cerebral and Cardiovascular Center, Suita, Osaka 5648565, Japan.; ^7^ The Institute of Medical Science, The University of Tokyo, Minato-ku, Tokyo 1088639 Japan.; ^8^ Center for Infectious Disease Education and Research (CiDER), Osaka University, Suita, Osaka 5650871 Japan.

## Abstract

Spermatozoa need to undergo an exocytotic event called the acrosome reaction before fusing with eggs. Although calcium ion (Ca^2+^) is essential for the acrosome reaction, its molecular mechanism remains unknown. Ferlin is a single transmembrane protein with multiple Ca^2+^-binding C2 domains, and there are six ferlins, dysferlin (DYSF), otoferlin (OTOF), myoferlin (MYOF), fer-1–like 4 (FER1L4), FER1L5, and FER1L6, in mammals. *Dysf*, *Otof*, and *Myof* knockout mice have been generated, and each knockout mouse line exhibited membrane fusion disorders such as muscular dystrophy in *Dysf*, deafness in *Otof*, and abnormal myogenesis in *Myof*. Here, by generating mutant mice of *Fer1l4*, *Fer1l5*, and *Fer1l6*, we found that only *Fer1l5* is required for male fertility. *Fer1l5* mutant spermatozoa could migrate in the female reproductive tract and reach eggs, but no acrosome reaction took place. Even a Ca^2+^ ionophore cannot induce the acrosome reaction in *Fer1l5* mutant spermatozoa. These results suggest that FER1L5 is the missing link between Ca^2+^ and the acrosome reaction.

## Teaser

FER1L5, a single transmembrane protein with multiple C2 domain, regulates Ca^2+^-activated exocytosis in spermatozoa.

## INTRODUCTION

Fertilization is a union of two gametes, spermatozoa, and oocytes. To fertilize eggs, spermatozoa need to travel a long distance in the female reproductive tract using flagella. During the migration, the acrosome located anterior to the nucleus in sperm heads needs to undergo an exocytotic event called the acrosome reaction to fertilize eggs (*1*, *2*). During the acrosome reaction, the outer acrosomal membrane fuses with the sperm plasma membrane and hydrolytic enzymes such as a serine protease (acrosin) (*3*, *4*), phospholipase A2 (*5*), and hyaluronidases (*6*) in the acrosome are exposed to facilitate fertilization. For hyaluronidases, hyaluronoglucosaminidase 5 (HYAL5) is localized in both plasma and acrosomal membranes of acrosome-intact spermatozoa and released during the acrosome reaction, while sperm adhesion molecule 1 (SPAM1), also called PH-20, is localized in the plasma membrane of acrosome-intact spermatozoa and retained after the acrosome reaction (*7*, *8*). Furthermore, izumo sperm-egg fusion 1 (IZUMO1), a type I transmembrane protein localized in the acrosomal membrane of acrosome-intact spermatozoa, relocates onto the cell surface after the acrosome reaction, which is a prerequisite for sperm-egg fusion (*9*).

As Ca^2+^ plays critical roles in the acrosome reaction, Ca^2+^ ionophores such as A23187 are potent inducers of the acrosome reaction. Several sperm proteins involved in Ca^2+^ influx were found, such as calcium channel, voltage-dependent, R type, alpha 1E subunit (Ca_v_2.3) (*10*); phospholipase C, delta 4 (PLCD4) (*11*); and transient receptor potential cation channel, subfamily C, member 2 (TRPC2) (*12*). Knockout (KO) mice of *Ca_v_2.3* or *Plcd4* result in impaired acrosome reaction in response to solubilized zona pellucida (ZP), the extracellular matrix surrounding the eggs (*10*, *11*). In contrast, A23187 could induce the acrosome reaction even in these mutant spermatozoa (*10*, *11*), indicating that these molecules play roles upstream of intracellular Ca^2+^ increase. The Ca^2+^-activated acrosome reaction is likely mediated by soluble *N*-ethylmaleimide–sensitive factor attachment protein receptor (SNARE) proteins that are involved in fusing vesicles with the target membrane (*13*, *14*). Still, the molecule that links Ca^2+^ increase and SNARE proteins in the acrosome reaction remains unknown.

Ferlin is a single transmembrane protein with multiple Ca^2+^-binding C2 domains and is involved in Ca^2+^-mediated membrane fusion. Ferlin was first found in *Caenorhabditis elegans*, in which mutations cause male infertility due to defective fusion of internal membranous organelles with the sperm plasma membrane (*15*). Misfire, a ferlin ortholog in *Drosophila melanogaster*, is also essential for male fertility, with mutations resulting in impaired sperm plasma membrane breakdown after fertilization (*16*). In mammals, there are six ferlin proteins: dysferlin (DYSF), otoferlin (OTOF), myoferlin (MYOF), fer-1–like 4 (FER1L4), FER1L5, and FER1L6. KO mice of *Dysf*, *Otof*, and *Myof* exhibit phenotypes related to impaired membrane fusion, such as muscular dystrophy in *Dysf* (*17*, *18*), deafness in *Otof* (*19*), and abnormal myogenesis in *Myof* (*20*). Disease-associated mutations in *DYSF* and *OTOF* were also found in humans (*21*–*23*). In contrast, it is unknown if ferlins play critical roles in male fertility in mammals.

This study generated *Fer1l4*, *Fer1l5*, and *Fer1l6* mutant mice and found that only *Fer1l5* is required for male fertility. Even A23187 cannot induce the acrosome reaction in *Fer1l5* mutant spermatozoa, suggesting that FER1L5 is indispensable for the Ca^2+^-activated fusion of plasma membrane and acrosomal membrane.

## RESULTS

### *Fer1l4*, *Fer1l5*, and *Fer1l6* are expressed in the mouse testis

**Fig. 1.**
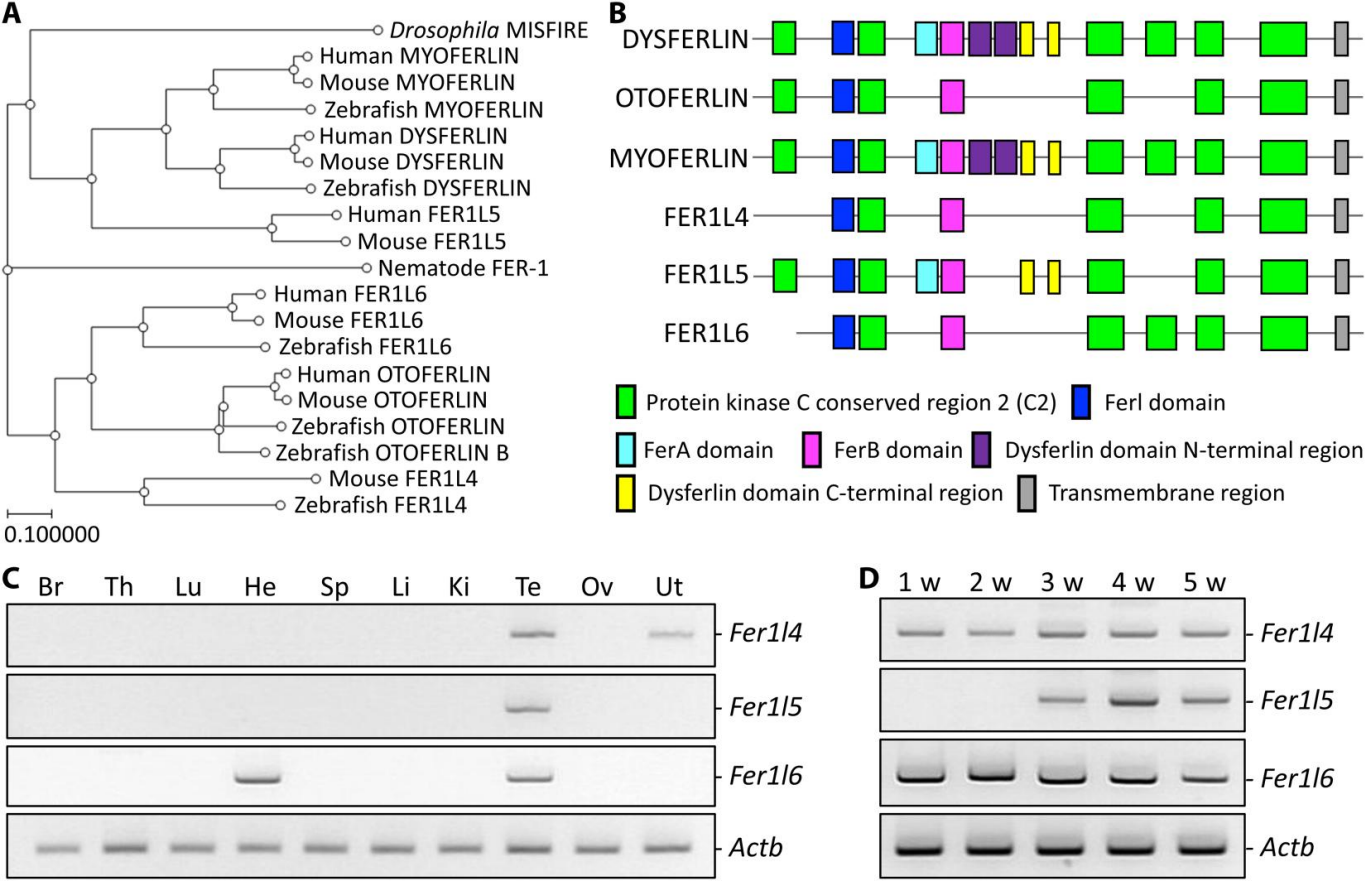
*Fer1l4*, *Fer1l5*, and *Fer1l6* are expressed in the testis. (**A**) Phylogenetic analysis of ferlin proteins in nematodes, *Drosophila*, zebrafish, mice, and humans. (**B**) Domain characteristics of mouse ferlin proteins. (**C**) RT-PCR of *Fer1l4*, *Fer1l5*, and *Fer1l6* using RNAs obtained from mouse tissues. *Actb* (actin beta) as control. Br, brain; Th, thymus; Lu, lung; He, heart; Sp, spleen; Li, liver; Ki, kidney; Te, testis; Ov, ovary; Ut, uterus. (**D**) RT-PCR of *Fer1l4*, *Fer1l5*, and *Fer1l6* using RNAs obtained from mouse postnatal testes. *Actb* as control.

Phylogenetic analyses of ferlin proteins in several model organisms are shown in Fig. 1A. There are six ferlin proteins, DYSF, OTOF, MYOF, FER1L4, FER1L5, and FER1L6, in mice. While DYSF, OTOF, MYOF, FER1L5, and FER1L6 are conserved, FER1L4 is missing in humans. We focused on *Fer1l4*, *Fer1l5*, and *Fer1l6* because their functions in vivo remain unclear. Consistent with other ferlins, FER1L4, FER1L5, and FER1L6 have a single transmembrane domain at the C terminus and several C2 domains that are thought to bind to Ca^2+^ and regulate Ca^2+^-dependent membrane targeting (Fig. 1B). We checked their expression in mouse tissues with reverse transcription polymerase chain reaction (RT-PCR) and found that *Fer1l4*, *Fer1l5*, and *Fer1l6* are expressed in the testis with additional *Fer1l4* expression in the uterus and *Fer1l6* expression in the heart (Fig. 1C). We performed RT-PCR using postnatal testis and found that *Fer1l4* and *Fer1l6* are expressed at week one, while *Fer1l5* starts to express at week three (Fig. 1D). These results suggest that *Fer1l4*, *Fer1l5*, and *Fer1l6* may play roles in male reproduction.

### *Fer1l5* is required for male fertility

**Fig. 2.**
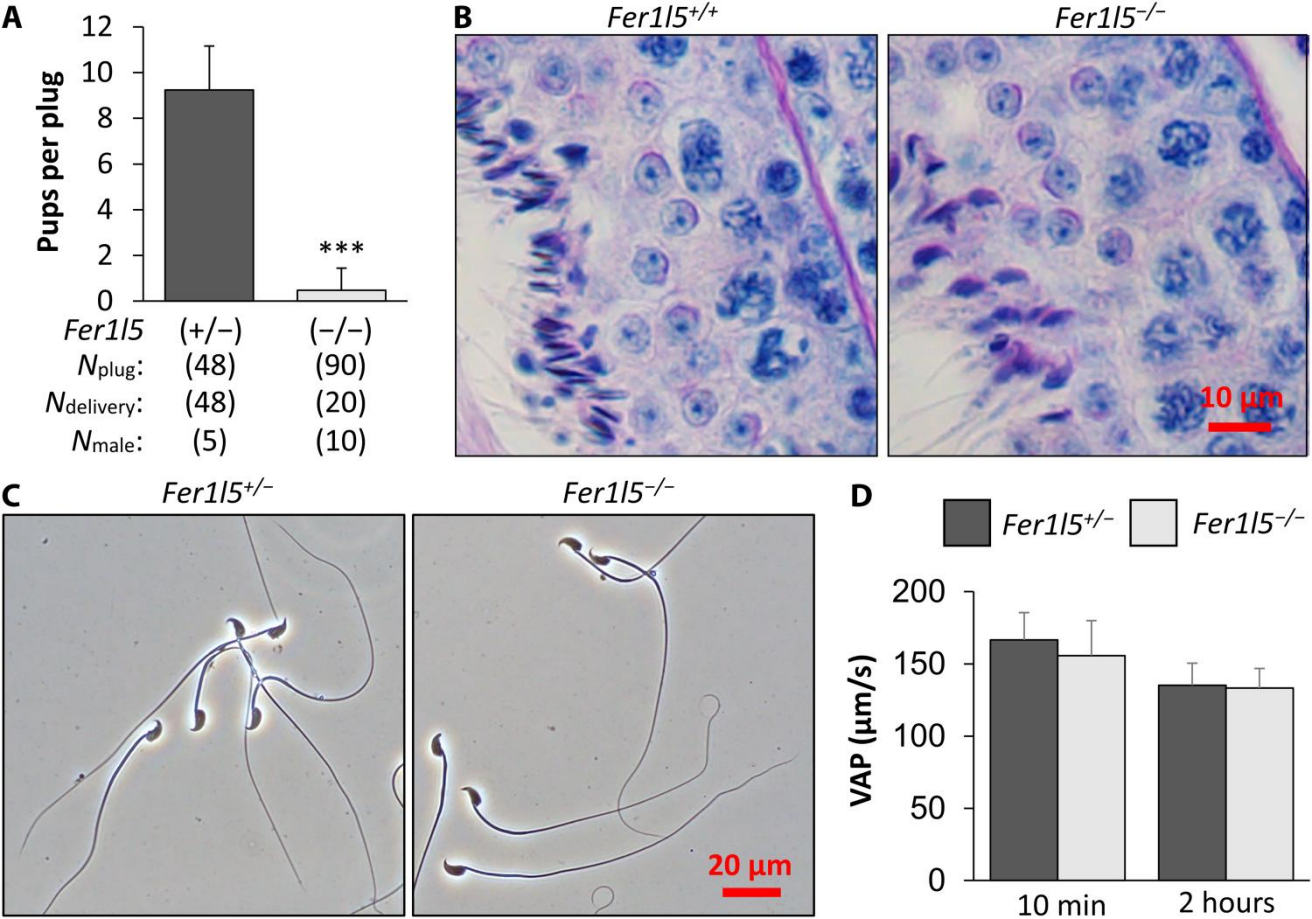
Fertility of *Fer1l5* mutant male mice is severely impaired. (**A**) Number of litters born per plug detected. ***P < 0.001 (Student’s *t* test). (**B**) Periodic acid–Schiff staining of testis sections. No overt abnormalities were found in *Fer1l5* mutant mice. (**C**) Observation of spermatozoa obtained from the cauda epididymis. No overt abnormalities were found in *Fer1l5* mutant spermatozoa. (**D**) Average path velocity (VAP) of sperm motility was analyzed. There were no significant differences. *n* = 7 males for the control and *n* = 9 males for *Fer1l5* mutant mice.

To analyze the function of *Fer1l4*, *Fer1l5*, and *Fer1l6* in vivo, we generated mutant mice for each gene. Exon 5 and 6 of *Fer1l4*, Exon 2 and 3 of *Fer1l5*, and Exon 4 and 5 of *Fer1l6* were deleted, which results in frameshift mutations (fig. S1, A to D). Each mutant mice were viable and had no gross abnormality in appearance or behavior. We then analyzed male fertility by mating each mutant male with wild-type females. While *Fer1l4* and *Fer1l6* mutant males were fertile (fig. S2A), the fertility of *Fer1l5* mutant males was severely impaired (Fig. 2A). Because both *Fer1l4* and *Fer1l6* lack the DYSF domain (Fig. 1B) and start expression from week one in the testis (Fig. 1D), the function of FER1L4 and FER1L6 may be similar in the testes and compensate each other. Therefore, we generated *Fer1l4* and *Fer1l6* double mutant mice by breeding, but the obtained males were still fertile (fig. S2A). We then focused on the analysis of *Fer1l5*. In contrast to male fertility, *Fer1l5* mutant females were fertile (fig. S2B). To ensure that *Fer1l5* is responsible for impaired male fertility, we generated transgenic (Tg) mice that expressed FLAG-tagged or PA-tagged *Fer1l5* under testis-specific calmegin (*Clgn*) promoter (fig. S2C). We detected FER1L5 in the testis of Tg mice with immunoblotting following immunoprecipitation (fig. S2, D and E) and confirmed that both *Fer1l5-FLAG* and *Fer1l5-PA* transgenes rescued impaired male fertility of *Fer1l5* mutant mice (fig. S2F).

### *Fer1l5* mutant spermatozoa are defective in ZP penetration and sperm-egg fusion

To clarify the cause of impaired male fertility of *Fer1l5* mutant mice, we analyzed testis and cauda epididymis sections, but no apparent abnormalities were found in *Fer1l5* mutant mice (Fig. 2B and fig. S3A). Furthermore, no differences were observed in sperm morphology (Fig. 2C) or motility parameters as analyzed by the computer-assisted sperm analysis system (Fig. 2D and fig. S3, B and C) between the control and *Fer1l5* mutant mice.

**Fig. 3.**
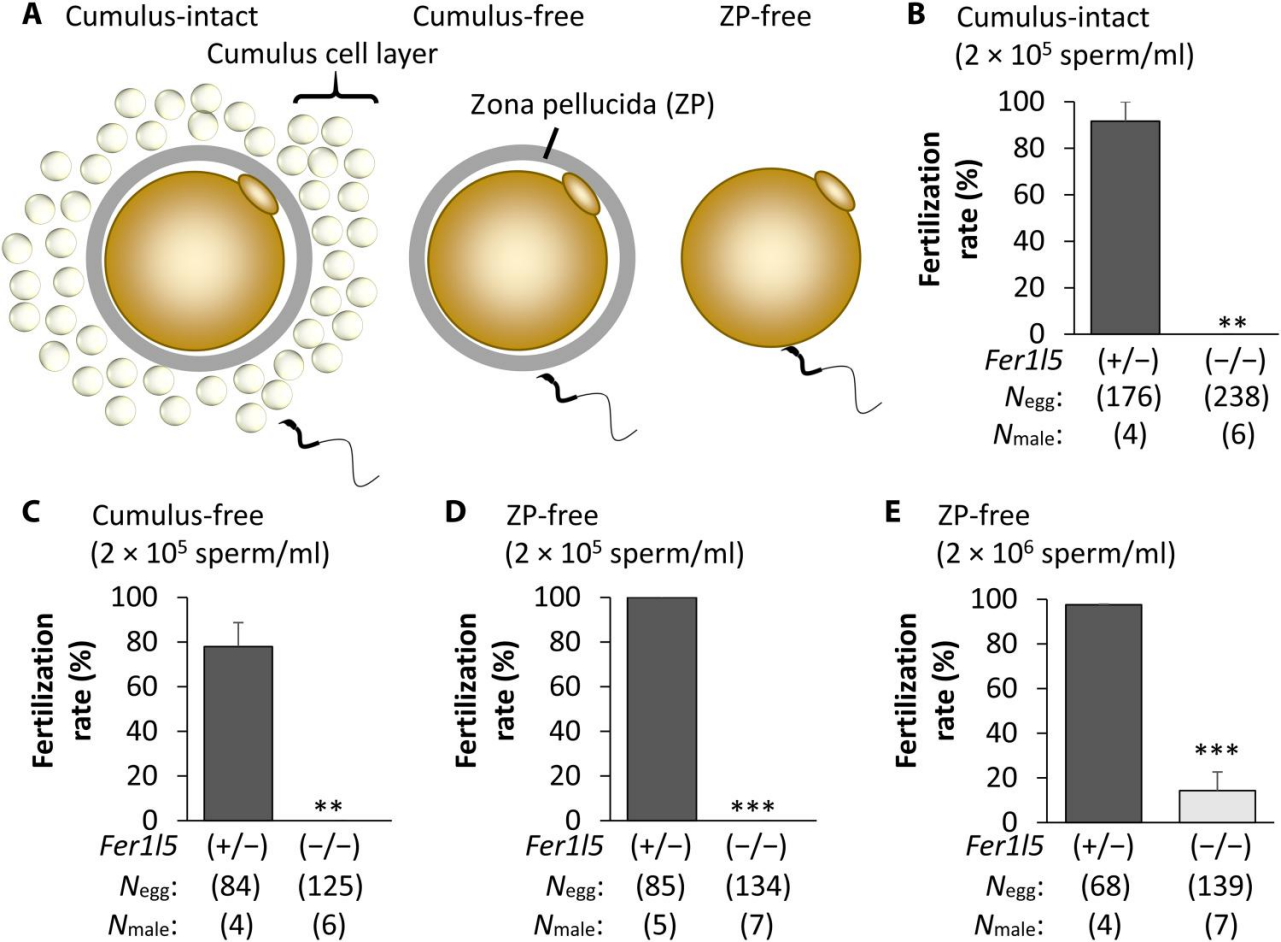
In vitro fertility of *Fer1l5* mutant male mice. (**A**) Cumulus-intact, cumulus-free, and ZP-free conditions were used to analyze in vitro fertility. (**B**) In vitro fertilization (IVF) with cumulus-intact oocytes. **P < 0.01 (Student’s *t* test). (**C**) IVF with cumulus-free oocytes. **P < 0.01 (Student’s *t* test). (**D**) IVF with ZP-free oocytes. ***P < 0.001 (Student’s *t* test). (**E**) IVF with ZP-free oocytes. Ten times more spermatozoa than in Fig. 3D were used for insemination. ***P < 0.001 (Student’s *t* test).

We then performed in vitro fertilization and found that *Fer1l5* mutant spermatozoa could not fertilize cumulus-intact eggs (Fig. 3, A and B), consistent with in vivo fertility results. Removing cumulus cells could not rescue impaired fertilization rates (Fig. 3C). Furthermore, even without the ZP, *Fer1l5* mutant spermatozoa could not fertilize eggs (Fig. 3D and fig. S4A). Notably, when we used 10 times more spermatozoa for insemination, no fertilization occurred with cumulus-intact or cumulus-free eggs in *Fer1l5* mutant mice (fig. S4B), but a few ZP-free eggs were fertilized (Fig. 3E and fig. S4A). These results indicate that *Fer1l5* mutant spermatozoa cannot fuse with eggs. We also did not observe any spermatozoa in the perivitelline space of ZP-intact eggs, which can be seen in the mutant spermatozoa lacking any known oocyte-fusing factors (*24*, *25*), suggesting that *Fer1l5* mutant spermatozoa have defects not only in fusing with eggs but also in penetrating the ZP.

### *Fer1l5* mutant spermatozoa cannot undergo the acrosome reaction in vitro

**Fig. 4.**
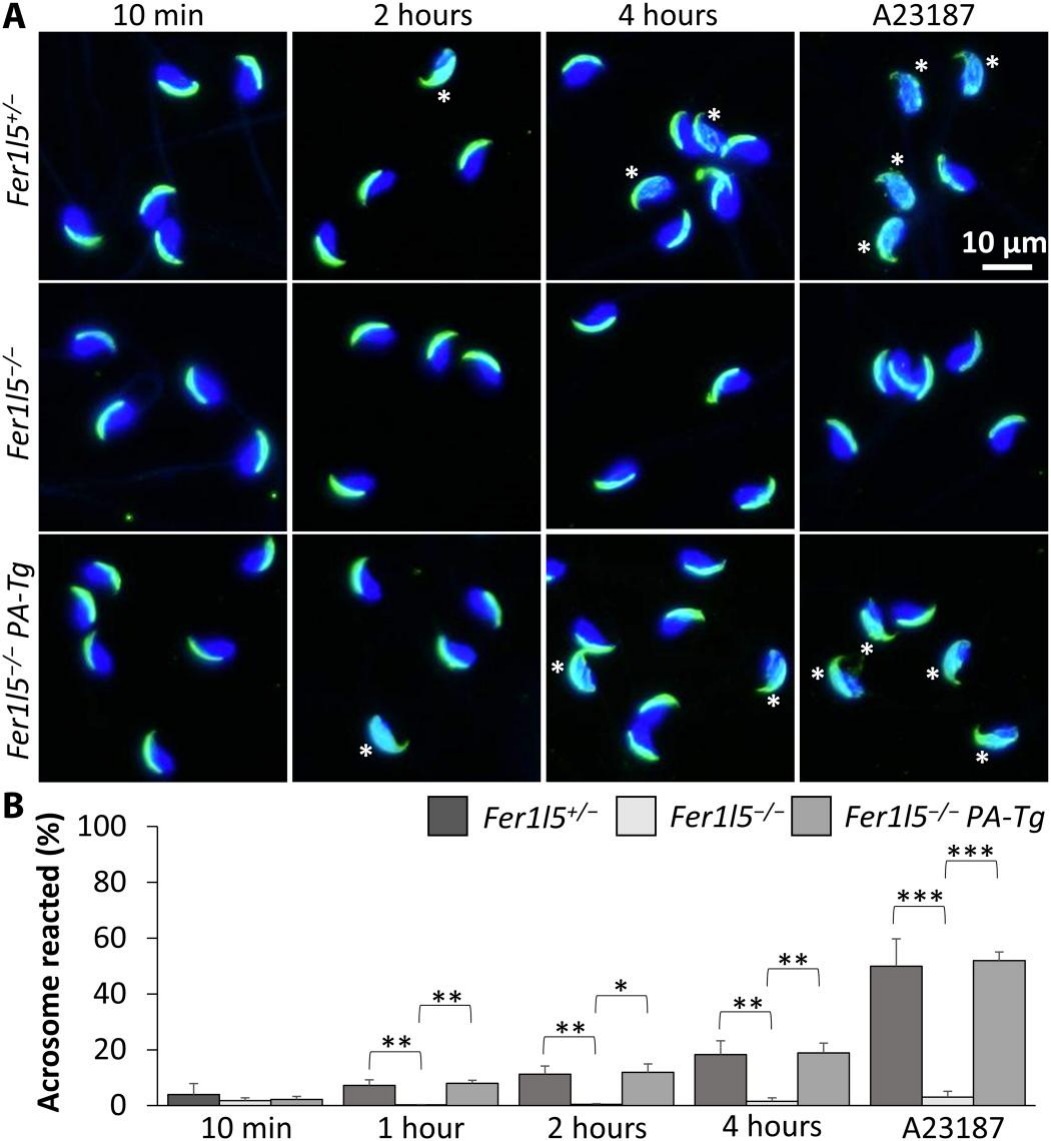
*Fer1l5* mutant spermatozoa cannot undergo the acrosome reaction in vitro. (**A**) Immunofluorescence staining for IZUMO1. IZUMO1 (green) relocates to the equatorial segment after the acrosome reaction (asterisks), which was used to analyze acrosome reaction rates. *Fer1l5* mutant spermatozoa rarely exhibit IZUMO1 relocation. Impaired acrosome reaction was rescued by *Fer1l5-PA* transgene. Nuclei are stained blue. (**B**) The acrosome reaction rates were analyzed by IZUMO1 staining after 10 min, 1 hour, 2 hours, and 4 hours incubation in capacitation medium. After 4 hours of incubation, Ca^2+^ ionophore A23187 was added to induce the acrosome reaction. *n* = 5 males for *Fer1l5^+/−^* mice and *n* = 3 males for *Fer1l5^−/−^* and *Fer1l5^−/−^ PA-Tg* mice. *P < 0.05, **P < 0.01, and ***P < 0.001 (Student’s *t* test).

Because it is widely accepted that only acrosome-reacted spermatozoa can penetrate the ZP and fuse with eggs, we analyzed the acrosome reaction in vitro by observing IZUMO1 that relocates onto the equatorial segment after the acrosome reaction (*9*). IZUMO1 was originally localized in the apical region (acrosomal cap) in both control and *Fer1l5* mutant spermatozoa (10 min after the collection from the cauda epididymis; Fig. 4A). After 1, 2, and 4 hours of incubation in capacitation medium, IZUMO1 relocates to the equatorial segment of the control spermatozoa; however, IZUMO1 stays in the apical region of almost all *Fer1l5* mutant spermatozoa (Fig. 4, A and B). Furthermore, even after A23187 treatment, almost no spermatozoa undergo the acrosome reaction in *Fer1l5* mutant mice (Fig. 4, A and B). Impairment of the acrosome reaction in *Fer1l5* mutant spermatozoa was rescued by PA-tagged *Fer1l5* transgene (Fig. 4, A and B), indicating that restored fertility in this line is due to the restored ability of spermatozoa to undergo the acrosome reaction.

Because immunostaining of IZUMO1 of fixed spermatozoa cannot tell the differences between live and dead spermatozoa, we also analyzed the acrosome reaction rates of live spermatozoa using Red Body Green Sperm (RBGS) Tg mice, whose spermatozoa lose the enhanced green fluorescent protein (EGFP) fluorescence signal after the acrosome reaction (*26*). Consistent with IZUMO1 staining results (Fig. 4, A and B), *Fer1l5* mutant spermatozoa could not undergo the acrosome reaction not only after 4 hours of incubation in capacitation medium but also after A23187 treatment (fig. S5A). These results indicate that FER1L5 is required for the acrosome reaction and functions downstream of Ca^2+^ influx.

### Tyrosine phosphorylation, hyperactivation, and acrosomal swelling occur in *Fer1l5* mutant spermatozoa

**Fig. 5.**
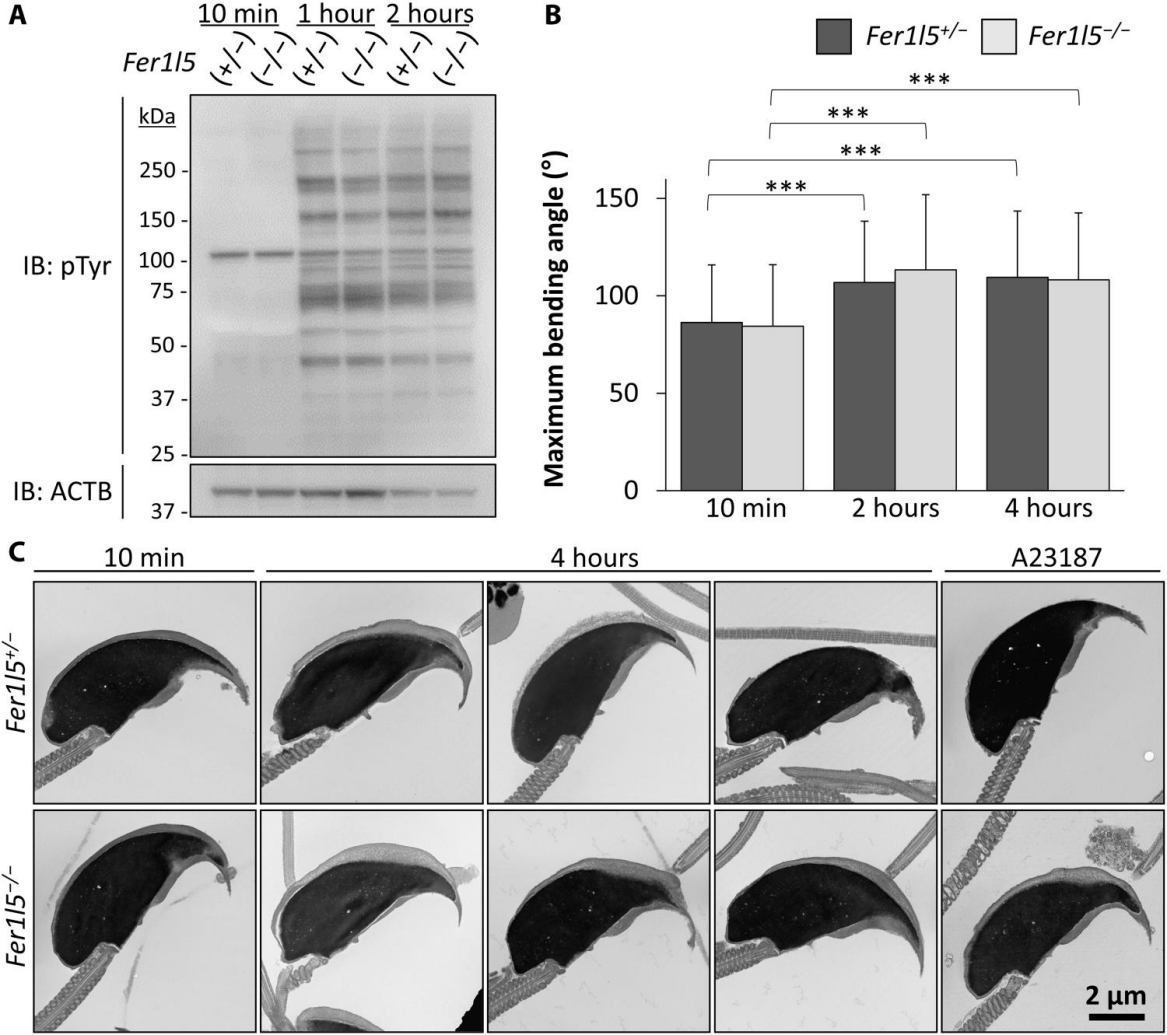
*Fer1l5* mutant spermatozoa can undergo capacitation-associated processes. (**A**) Immunoblot (IB) analysis for phosphotyrosine (pTyr) in noncapacitated (10 min) and capacitated (1 and 2 hours) spermatozoa. ACTB (actin beta) as control. (**B**) Maximal bending angles of the midpiece in primary antihook curvature were measured. We analyzed 60 spermatozoa from three mice of each genotype (20 spermatozoa from each mouse). No significant differences were found between the control and *Fer1l5* mutant mice. ***P < 0.001 (Student’s *t* test). (**C**) Observation of sperm heads with transmission electron microscopy. Spermatozoa were incubated 10 min or 4 hours in capacitation medium, and A23187 was added after 4 hours of incubation. The acrosome swelling was observed in *Fer1l5* mutant spermatozoa as well.

Capacitation is the biochemical process that spermatozoa undergo after ejaculation into the female reproductive tract and is thought to be a prerequisite for the acrosome reaction (*27*). Therefore, we observed tyrosine phosphorylation, a hallmark of the capacitation. After 1 and 2 hours of incubation in capacitation medium, enhanced tyrosine phosphorylation signals were observed in both the control and *Fer1l5* mutant spermatozoa (Fig. 5A), suggesting that the signal transduction pathways that induce tyrosine phosphorylation were not impaired.

Next, we checked the hyperactivation of sperm flagella, which is important for penetrating the ZP (*28*, *29*). Hyperactivation occurs during capacitation in which the spermatozoa exhibit a high amplitude and asymmetrical beating pattern of flagella (*28*, *29*). We examined the maximal bending angle of the midpiece in primary antihook curvature, which is used to quantitatively analyze hyperactivation (*30*). Consistent with the control spermatozoa, the maximum bending angles increased in *Fer1l5* mutant spermatozoa after 2 and 4 hours of incubation in capacitation medium (Fig. 5B), suggesting that *Fer1l5* mutant spermatozoa could undergo hyperactivation without the acrosome reaction.

Using transmission electron microscopy, we also observed the acrosome swelling that occurs during the capacitation process and is thought to be required for the fusion of the plasma membrane and the outer acrosomal membrane during the acrosome reaction (*31*). After 10 min of incubation in capacitation medium, no abnormalities were observed in the acrosomal morphology in *Fer1l5* mutant spermatozoa (Fig. 5C). After 4 hours of incubation, acrosome swelling was observed in both control and *Fer1l5* mutant spermatozoa; however, the acrosome reaction did not occur in the *Fer1l5* mutant spermatozoa (Fig. 5C). These results indicate that *Fer1l5* mutant spermatozoa can undergo capacitation-associated processes such as tyrosine phosphorylation, hyperactivation, and acrosomal swelling, but no fusion between the plasma membrane and outer acrosomal membrane takes place.

### FER1L5 interacts with SNARE proteins

**Fig. 6.**
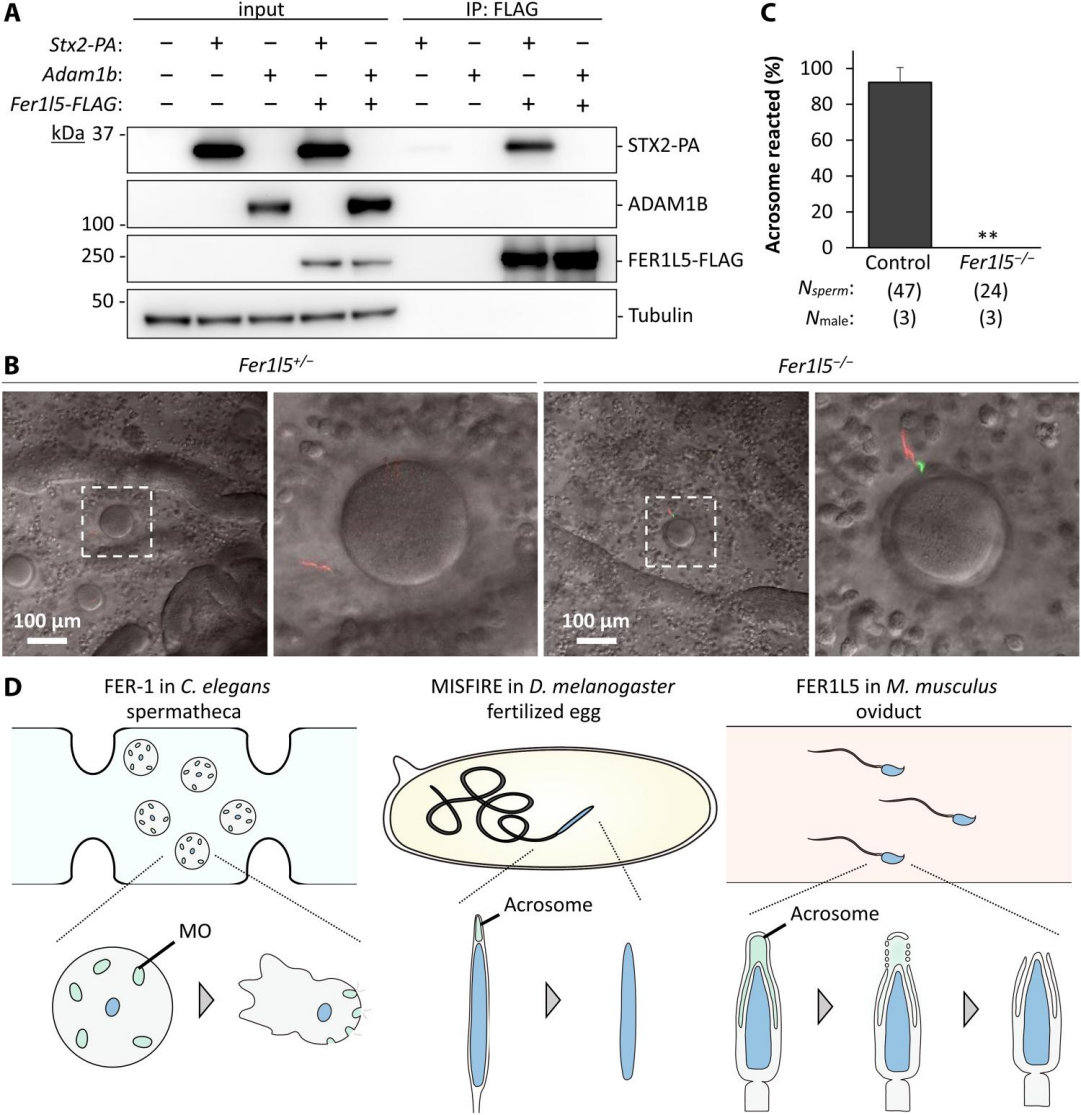
*Fer1l5* mutant spermatozoa cannot undergo the acrosome reaction in vivo. (**A**) Immunoprecipitation (IP) of FER1L5-FLAG using anti-FLAG antibody. *Fer1l5-FLAG*, *Stx2-PA*, and *Adam1b* were transiently expressed in HEK293T cells. FER1L5 interacts with STX2-PA but not ADAM1B. Tubulin as control. (**B**) Observation of spermatozoa in the ampulla 20 hours after human chorionic gonadotropin (hCG) injection. The midpiece exhibited *Discosoma* sp. Red 2 (DsRed2) signal (red) and the intact acrosome exhibited EGFP (green). The *Fer1l5* mutant spermatozoa was acrosome intact with EGFP signal. (**C**) Percentages of acrosome-reacted spermatozoa in the ampulla. *Fer1l5* mutant spermatozoa were observed in the ampulla but were not acrosome-reacted. (**D**) Function of ferlins in spermatozoa of *C. elegans*, *D. melanogaster*, and *Mus musculus*. In *C. elegans*, FER-1 is involved in the fusion of membranous organelle (MO) with the plasma membrane, which activates a morphological change from a quiescent, round spermatid to a motile, amoeboid spermatozoa. In *D. melanogaster*, MISFIRE regulates sperm plasma membrane breakdown after sperm entry into oocytes for the fusion of male and female pronuclei. In *M. musculus*, FER1L5 regulates the acrosome reaction in the oviduct.

It is known that OTOF, a ferlin that is essential for hearing, interacts with SNARE proteins such as syntaxin 1 (STX1) and regulates Ca^2+^-dependent SNARE-mediated membrane fusion (*19*, *32*). Furthermore, SNARE proteins are thought to regulate membrane fusion during the acrosome reaction (*13*, *14*). Because these studies suggest an interaction between ferlins and syntaxins, we focused on STX2, a protein that was suggested to be involved in the acrosome reaction (*13*). It has been reported that *Stx2* mutant male mice were infertile because of the absence of round spermatids and mature spermatozoa, confirming that STX2 plays a role in the testis (*33*). However, this phenotype makes it difficult to analyze the acrosome reaction in the *Stx2* mutant mice because of the lack of spermatozoa. To uncover the relationship, we coexpressed FLAG-tagged FER1L5 and PA-tagged STX2 in human embryonic kidney 293T (HEK293T) cells and performed an immunoprecipitation analysis using an anti-FLAG antibody (Fig. 6A). After immunoblotting, we found that FER1L5 interacts with STX2 (Fig. 6A). In contrast, FER1L5 did not interact with negative control, a disintegrin and metallopeptidase domain 1b (ADAM1B), which is localized in the sperm plasma membrane (Fig. 6A) (*34*). These results suggest that FER1L5 may regulate SNARE-mediated membrane fusion during the acrosome reaction.

### *Fer1l5* mutant spermatozoa can reach the eggs but are not acrosome-reacted in vivo

Last, we observed sperm migration in the female reproductive tract using RBGS Tg mice to analyze the acrosomal status in vivo. We observed spermatozoa in the female reproductive tract 2 and 8 hours after observing vaginal plugs [equivalent to 14 and 20 hours after human chorionic gonadotropin (hCG) injection]. *Fer1l5* mutant spermatozoa could pass through the utero-tubal junction (UTJ) that connect the uterus and oviduct (fig. S5B) and reach the ZP surrounding the eggs in the ampulla (Fig. 6B), indicating that the acrosome reaction is not essential for spermatozoa to migrate in the female reproductive tract and pass through the UTJ and the layer of cumulus cells. Although *Fer1l5* mutant spermatozoa could reach the eggs, no spermatozoa were acrosome-reacted (Fig. 6, B and C). These results indicate that FER1L5 is required for the acrosome reaction and penetrating the ZP in vivo, consistent with the analyses in vitro.

## DISCUSSION

In this study, we reveal that FER1L5 regulates the acrosome reaction in mice, similar to FER-1 regulating the membranous organelle fusion in *C. elegans* and MISFIRE regulating the sperm plasma membrane breakdown in *D. melanogaster*. These results indicate that ferlin function in spermatozoa is evolutionarily conserved, although vesicle fusion during fertilization takes place at different times. In *C. elegans*, vesicle fusion occurs during spermatid activation (*15*), while in *D. melanogaster*, it occurs during plasma membrane breakdown after sperm entry into the oocytes (*16*). In mice, fusion occurs in the acrosome reaction during sperm migration in the female reproductive tract (Fig. 6D) (*1*, *2*). Although vesicle fusion takes place at different times, these processes may be regulated by similar molecular mechanisms through ferlins. Intriguingly, *Fer1l5* was not found in zebrafish, in which spermatozoa lack the acrosome (Fig. 1A).

In *C. elegans*, it has been reported that FER-1 is localized in the membranous organelles (*35*). It is tempting to speculate that FER1L5 is localized in the acrosomal membrane like FER-1 in the membrane organelles, but we could not obtain an anti-FER1L5 antibody that worked for immunoblotting or immunofluorescence to analyze its localization. In *Fer1l5-FLAG* or *Fer1l5-PA* Tg mice, we detected FER1L5-FLAG or FER1L5-PA using large amounts of testicular proteins, which hamper the analysis of FER1L5 localization. Therefore, we cannot rule out the possibility that FER1L5 plays roles in the testes and impaired acrosome reaction in *Fer1l5* mutant mice is a consequence of defective spermatogenesis; however, previous proteomics analysis showed that mature spermatozoa contained FER1L5 in several mammalian species such as humans (*36*), cattle (*37*), yaks (*38*), and rams (*39*). Furthermore, our mass spectrometry (MS) analyses detected FER1L5 peptides in mouse mature spermatozoa (fig. S6, A to C), indicating that FER1L5 may play roles in mature spermatozoa.

Using heterologous expression system, we found that FER1L5 could interact with syntaxin consistent with OTOF (*19*, *32*), suggesting that FER1L5 may regulate Ca^2+^-dependent SNARE-mediated vesicle fusion during the acrosome reaction. This idea is supported by the result that even a Ca^2+^ ionophore cannot induce the acrosome reaction in *Fer1l5* mutant spermatozoa. Furthermore, recent in silico structural modeling predicted that the C2 domain of human FER1L5 could bind to Ca^2+^ (*40*), suggesting that FER1L5 may functions as a calcium sensor that regulates the acrosome reaction. It has been reported that OTOF acts as a calcium sensor that regulates synaptic vesicle exocytosis in cochlear hair cells (*32*).

*Fer1l5* mutant mice can be a good model to study the acrosome reaction. In this study, we found that *Fer1l5* mutant spermatozoa could undergo hyperactivation (Fig. 5B), suggesting that the acrosome reaction is not required for hyperactivation. Furthermore, we confirmed that the acrosome-intact spermatozoa could reach the ZP in vivo (Fig. 6B). Because the acrosome reaction is severely impaired, *Fer1l5* mutant spermatozoa could not fertilize cumulus-intact eggs in vitro even with a higher concentration of spermatozoa (fig. S4B). In contrast, a few pups were obtained from *Fer1l5* mutant males in vivo (Fig. 2A). These results suggest that the acrosome reaction is induced more efficiently in vivo than in vitro. Considering that A23187 rarely induced the acrosome reaction in *Fer1l5* mutant mice, Ca*^2+^*-dependent signaling pathway and other pathways such as mechanical stimulus (*41*) may be involved in inducing the acrosome reaction in vivo.

In summary, our results indicate that FER1L5 is important for the acrosome reaction and male fertility in mice. Identification of FER1L5 as a key molecule of Ca^2+^-activated acrosome reaction will shed light on the molecular mechanism of the acrosome reaction. Furthermore, human *FER1L5* exhibits testis-enriched expression (*42*), and its function may be conserved in humans as well. Revealing FER1L5-associated molecules and their functions may help us understand the etiology of human male infertility.

## MATERIALS AND METHODS

### Animals

All mice used in this study were purchased from CLEA Japan (Tokyo, Japan) or Japan SLC (Shizuoka, Japan). Mice were maintained under specific pathogen–free conditions in a temperature-controlled environment, 12-hour light/12-hour dark cycle, and ad libitum feeding. All animal experiments were approved by the Animal Care and Use Committee of the Research Institute for Microbial Diseases, Osaka University (#Biken-AP-H30-01) and were conducted in accordance with the guidelines established by the Research Institute for Microbial Diseases, Osaka University.

### Generation of *Fer1l4*, *Fer1l5*, and *Fer1l6* mutant mice

The targeting vectors were obtained from the International Mouse Phenotyping Consortium (*Fer1l6*) (www.mousephenotype.org/) (*43*) or generated using pNT1.1 (*Fer1l4* and *Fer1l5*) (www.ncbi.nlm.nih.gov/nuccore/378747675) (*44*). The primers used to amplify long arms and short arms for pNT1.1 are listed in table S1. The vectors were electroporated into embryonic stem (ES) cells (EGR-G101 for generating *Fer1l4* mutant mice and EGR-G01 for generating *Fer1l5* and *Fer1l6* mutant mice) (*45*) after linearization, and potentially targeted ES cell clones were selected with G418 (150 μg/ml; Thermo Fisher Scientific, Waltham, MA, USA). The homologously recombined ES cells were then injected into Institute of Cancer Research (ICR) eight-cell embryos. The embryos were cultivated in a potassium simplex optimized medium (KSOM) (*46*) until the next day and transferred into pseudo-pregnant females. The obtained chimera mice were mated with B6D2F1 females for germline transmission. For *Fer1l6*, floxed mice were further mated with B6D2 *CAG-Cre* Tg mice (*47*).

### Generation of *Fer1l5*-FLAG and *Fer1l5*-PA Tg mice

Mouse *Fer1l5* cDNA-FLAG tag or *Fer1l5* cDNA-PA tag with a rabbit polyadenylate [poly(A)] signal under the mouse *Clgn* promoter (fig. S2C) was prepared. The linearized DNA was injected into one of the pronuclei of fertilized eggs that were obtained by the mating of superovulated B6D2F1 females and B6D2F1 males, and the zygotes were cultivated in KSOM to two-cell stage and then transferred into pseudo-pregnant females.

### Cell culture

HEK293T cells (*48*) were cultured in Dulbecco’s modified Eagle’s medium (Thermo Fisher Scientific) supplemented with 10% fetal bovine serum (Biowest, Nuaille, France) and 1% penicillin-streptomycin-glutamine (Thermo Fisher Scientific) at 37°C under 5% CO_2_.

### Phylogenetic analysis

The phylogenetic trees of candidate genes were created using the neighbor-joining method with the GENETYX software (GENETYX, Tokyo, Japan). Amino acid sequences of each protein were obtained from the National Center for Biotechnology Information Entrez Protein database. The accession numbers of each amino acid sequence used were as follows: *Drosophila* MISFIRE (NP_001137919.1), human MYOF (NP_038479.1), mouse MYOF (NP_001093104), zebrafish MYOF (NP_957169.2), human DYSF (NP_001124459.1), mouse DYSF (NP_067444.2), zebrafish DYSF (XP_005172350.1), human FER1L5 (NP_001280012.1), mouse FER1L5 (NP_001264005.1), nematode FER-1 (NP_492337.1), human FER1L6 (NP_001034201.2), mouse FER1L6 (NP_001357841.1), zebrafish FER1L6 (XP_021334459.1), human OTOF (NP_919224.1), mouse OTOF (NP_114081.2), zebrafish OTOF (NP_001025283.1), zebrafish OTOFB (XP_017207363.1), mouse FER1L4 (NP_001130028.1), and zebrafish FER1L4 (XP_021335523.1).

### Reverse transcription polymerase chain reaction

Mouse RNAs were prepared from multiple adult tissues of C57BL/6N or testes from 1- to 5-week-old males using TRIzol (Thermo Fisher Scientific, Waltham, MA, USA). The obtained RNA was immediately reverse-transcribed to cDNA with SuperScript IV First-Strand Synthesis System (Thermo Fisher Scientific) using oligo(dT) as primers. PCR primers for each gene are shown in table S1.

### Mating tests

Sexually matured male mice were individually caged with two 8-week-old B6D2F1 female mice for 2 months, and the number of pups was counted. For *Fer1l5* mutant mice, plugs were checked every morning.

### Histological analysis of testes and cauda epididymides

Male mice (12 to 18 weeks old) were euthanized, and testes and cauda epididymides were dissected. Testes and cauda epididymides were fixed overnight at 4°C in Bouin’s fluid (Polysciences Inc., Warrington, PA, USA), embedded in paraffin, and sectioned at a thickness of 5 μm. Paraffin sections were then rehydrated, treated with 1% periodic acid for 20 min at room temperature, and incubated with Schiff’s reagent (FUJIFILM Wako Pure Chemical, Osaka, Japan) for 20 min at room temperature. The sections were stained with Mayer’s hematoxylin solution (FUJIFILM Wako Pure Chemical) and observed using a BX-53 microscope (Olympus, Tokyo, Japan).

### Morphological observation of spermatozoa

Spermatozoa were collected from the cauda epididymis and dispersed in phosphate-buffered saline (PBS) (Thermo Fisher Scientific). The spermatozoa were observed using a BX-53 microscope (Olympus).

### Sperm motility analysis

Spermatozoa obtained from the cauda epididymis were incubated in a drop of Toyoda Yokoyama Hosi (TYH) medium (*1*) at 37°C under 5% CO_2_. Ten minutes, 2 hours, or 4 hours after incubation, spermatozoa were collected from the top of the drop. Sperm motility was analyzed using the CEROS sperm analysis system (Hamilton Thorne Biosciences, Beverly, MA, USA). Analysis settings were used as described previously (*29*, *49*). For analyzing maximum bending angles, spermatozoa were observed with an Olympus BX-53 microscope equipped with a high-speed camera (HAS-L1, Ditect, Tokyo, Japan).

### In vitro fertilization

Spermatozoa collected from the cauda epididymis were incubated in a drop of TYH medium for 2 hours at 37°C under 5% CO_2_. For collecting eggs, Center for Animal Resources and Development (CARD) HyperOva (0.1 ml; Kyudo, Saga, Japan) was injected into the abdominal cavity of B6D2F1 female mice, followed by hCG (five units, ASKA Pharmaceutical, Tokyo, Japan) with 48-hour interval. Eggs were then collected from the superovulated females, treated with hyaluronidase (330 μg/ml; Sigma-Aldrich, St. Louis, MO, USA) for 10 min to remove the cumulus cells (cumulus-free eggs) or with collagenase (1 mg/ml; Sigma-Aldrich) for 10 min to remove the ZP (ZP-free eggs). The spermatozoa were added to the TYH drops that contain intact, cumulus-free, or ZP-free eggs at a final density of 2 × 10^5^ or 2 × 10^6^ spermatozoa/ml and incubated at 37°C under 5% CO_2_. The formation of pronuclei was observed 6 hours after insemination for ZP-free eggs. Two-cell embryos were counted the next day for intact or cumulus-free eggs.

### Analysis of the acrosome reaction rates in vitro with IZUMO1 staining

Cauda epididymal spermatozoa were dispersed in a drop of TYH medium for 10 min, 1 hour, 2 hours, or 4 hours of incubation at 37°C under 5% CO_2_. To induce the acrosome reaction with Ca^2+^ ionophore, 20 μM A23187 (Merck, Darmstadt, Germany) was added after 4 hours of incubation. The spermatozoa were spotted onto slides, air-dried, fixed with 4% paraformaldehyde for 10 min, and washed in PBS for 5 min. The slides were blocked with PBS containing 5% bovine serum albumin and 10% goat serum for 1 hour at room temperature. The slides were incubated with anti-IZUMO1 antibody (1:500) (*50*) overnight at 4°C, washed with PBS three times for 10 min each, and incubated with Alexa Fluor 488–conjugated secondary antibody (1:200; Thermo Fisher Scientific) at room temperature for 2 hours. The slides were washed with PBS three times for 10 min each, incubated with Hoechst 33342 (2 μg/ml) (Thermo Fisher Scientific) for 15 min, and washed with PBS three times for 10 min each. Slides were observed with an Olympus BX-53 microscope. The acrosome reaction rates were analyzed by counting the number of spermatozoa with IZUMO1 in the equatorial segment as acrosome reacted. More than 600 spermatozoa were counted for each trial.

### Analysis of the acrosome reaction rates in vitro using RBGS mice

*Fer1l5* mutant mice were crossed with B6D2 Tg mice carrying *CAG/Su9-DsRed2*, *Acr3-EGFP* (RBGS) (*26*). Cauda epididymal spermatozoa were dispersed in a drop of TYH medium for 10 min or 4 hours of incubation at 37°C under 5% CO_2_. To induce the acrosome reaction with Ca^2+^ ionophore, 20 μM A23187 (Merck) was added after 4 hours of incubation. Propidium iodide (PI) (10 μg/ml) was added to the drop, and an aliquot of the sperm suspension was placed on a glass slide. Acrosome reaction rates were determined by observing EGFP signals while distinguishing viable cells with PI staining with a BX-53 microscope (Olympus). Spermatozoa with EGFP signals in the acrosome were counted as acrosome intact. More than 200 spermatozoa were counted for each trial.

### Observation of sperm migration and analysis of the acrosome reaction rates in vivo

B6D2F1 female mice were superovulated as mentioned above and mated with male mice that contained the RBGS transgene. Two hours after observation of vaginal plugs or 20 hours after hCG injection, female reproductive tracts were dissected and spermatozoa inside the oviduct or ampulla were observed. To confirm that the spermatozoa were alive, motile spermatozoa were observed using a Nikon Eclipse Ti microscope connected to a Nikon C2 confocal module (Nikon, Tokyo, Japan). Spermatozoa with EGFP signals in the acrosome were counted as acrosome intact.

### Observation of the acrosome swelling

Cauda epididymal spermatozoa were dispersed in a drop of TYH medium for 10 min or 4 hours of incubation at 37°C under 5% CO_2_. To induce the acrosome reaction with a Ca^2+^ ionophore, 20 μM A23187 (Merck) was added after 4 hours of incubation. The spermatozoa were washed once with cold PBS and then with electron microscope (EM) buffer [30 mM Hepes (pH 7.8), 100 mM NaCl, and 2 mM CaCl_2_]. The samples were fixed with 1% glutaraldehyde in EM buffer for 1 hour at 4°C and washed once with EM buffer. Spermatozoa with a final concentration of 2 × 10^6^ spermatozoa/ml in EM buffer were spotted onto a cover slip coated with 0.001% poly-l-lysine using Cytospin 4 (1000 rpm for 3 min) (Thermo Fisher Scientific). The samples were washed three times for 5 min each in 0.1 M phosphate buffer (pH 7.4) containing 4% sucrose and postfixed in 1% OsO_4_ and 0.5% potassium ferrocyanide of 0.1 M phosphate buffer (pH 7.4) for 60 min at room temperature. After washing in distilled water, samples were dehydrated in a graded series of ethanol solutions: 50, 70, and 90% ethanol on ice and three times in 100% ethanol for 10 min at room temperature. Dehydrated samples were incubated in 50% epoxy resin mixture dissolved in 100% ethanol (ethanol:resin = 1:1) for 60 min at room temperature. Samples were incubated twice in pure epoxy resin mixture for 60 min each at room temperature and embedded in epoxy resin for 2 days at 60°C. Ultrathin sections (80 nm in thickness) were obtained and stained with 8% uranyl acetate solution for 30 min, briefly washed three times in distilled water, stained with lead staining solution for 2 min, and washed three times in distilled water. The ultrathin sections were observed using a JEM-1400 plus EM (JEOL, Tokyo, Japan) at 80 kV with a charge-coupled device Veleta 2K × 2K camera (Olympus).

### Immunoblotting

For analyzing tyrosine phosphorylation, spermatozoa were collected from the cauda epididymis and incubated in TYH medium for 10 min, 1 hour, or 2 hours. The spermatozoa were then lysed with a sample buffer (66 mM tris-HCl, 2% SDS, 10% glycerol, and 0.005% bromophenol blue) and centrifuged at 15,300*g* for 15 min at 4°C to collect supernatants. Samples were subjected to SDS–polyacrylamide gel electrophoresis (PAGE) under reduced conditions and transferred to polyvinylidene difluoride membrane using the Trans-Blot Turbo system (Bio-Rad, Foster City, CA, USA). The blots were blocked with 10% skimmed milk or Blocking One (#03953-95, Nacalai Tesque, Kyoto, Japan), incubated with primary antibodies [actin beta (ACTB), 1:1000; ADAM1B, 1:1000; FLAG PM020, 1:500; PA, 1:1000; phosphotyrosine, 1:1000; tubulin, 1:1000] overnight at 4°C, and incubated with secondary antibodies conjugated to horseradish peroxidase (1:5000) for 2 hours at room temperature. The antibodies used can be found in table S2. The signals were detected with Chemi-Lumi One Super (#02230, Nacalai Tesque) or Chemi-Lumi One Ultra (#11644, Nacalai Tesque).

### Collection of testis lysates for immunoprecipitation

Testes were lysed with a solution containing 1% Triton X-100, 50 mM NaCl, 20 mM tris-HCl (pH 7.4), and protease inhibitor mixture (Nacalai Tesque); incubated for 1 hour at 4°C; and centrifuged at 15,300*g* for 15 min at 4°C to collect the supernatants.

### Generation of recombinant proteins for immunoprecipitation

cDNAs encoding *Fer1l5*, *Stx2*, and *Adam1b* were amplified from mouse testis (C57BL/6N) and cloned into FLAG-tagged (C terminus), PA-tagged (C terminus), and untagged pCAG vectors, respectively, that contain the cytomegalovirus immediate enhancer/β-actin (CAG) promoter and a rabbit globin poly(A) signal (*51*). The expression vectors were transfected transiently into HEK293T cells, and the cells were cultured for 24 hours before collection. The cells were then lysed with a solution containing 1% Triton X-100, 50 mM NaCl, 20 mM tris-HCl (pH 7.4), and protease inhibitor mixture (Nacalai Tesque); incubated for 30 min at 4°C; and centrifuged at 15,300*g* for 15 min at 4°C to collect supernatants.

### Immunoprecipitation

The protein lysates were incubated for 60 min at 4°C with anti-FLAG antibody (M2, Sigma-Aldrich) or anti-PA antibody (FUJIFILM Wako Pure Chemical)–conjugated Dynabeads (Thermo Fisher Scientific). The complexes were washed three times with a solution containing 40 mM tris-HCl, 150 mM NaCl, 0.1% Triton X-100, and 10% glycerol and eluted with a sample buffer. Immunoblotting with collected samples were performed as mentioned above.

### Mass spectrometry analysis

Spermatozoa collected from the cauda epididymis were lysed with a solution containing 1% Triton X-100, 50 mM NaCl, 20 mM tris-HCl (pH 7.4), and protease inhibitor mixture (Nacalai Tesque); incubated for 2 hours at 4°C; and centrifuged at 15,300*g* for 15 min at 4°C to collect supernatants. The samples were subjected to MS analyses directly or after SDS-PAGE under reduced condition. To visualize proteins in gels, silver staining was performed following the manufacturer’s protocol (#05773-11, Nacalai Tesque). Mass spectrometry analyses were performed as described previously with some modifications (*52*). Briefly, peptides were subjected to nanocapillary reversed phase liquid chromatography with tandem MS (LC-MS/MS) analysis using a C18 column on a NanoLC System (Bruker Daltoniks, Bremen, Germany) connected to a timsTOF Pro mass spectrometer (Bruker Daltoniks) and a modified nanoelectrospray ion source (CaptiveSpray, Bruker Daltoniks). The mobile phase consisted of water containing 0.1% formic acid (solvent A) and acetonitrile containing 0.1% formic acid (solvent B) at linear gradient elution. The ion spray voltage was set in the positive ion mode. Ions were collected in the trapped ion mobility spectrometry (TIMS) device. During the collection of MS/MS data, the TIMS cycle included parallel accumulation serial fragmentation–MS/MS scans (*53*), and nitrogen gas was used as collision gas. Proteins were identified using MASCOT (version 2.7.0, Matrix Science, London, UK) against the UniProt database. Scaffold (version Scaffold_5.0.1, Proteome Software Inc., Portland, OR, USA) was used to validate peptide and protein identifications. Peptide identification probability and protein identification probability were calculated using the PeptideProphet algorithm (*54*) and ProteinProphet algorithm (*55*), respectively.

### Statistical analysis

A two-tailed unpaired Student’s *t* test was performed using Excel (Microsoft, Redmond, WA, USA). Significant differences were considered at *P < 0.05, **P < 0.01, and ***P < 0.001. Data represent the means ± SD.

## Notes

View/request a protocol for this paper from *Bio-protocol*.
