# Supplementary material for: Testis-enriched ferlin, FER1L5, is required for Ca2+-activated acrosome reaction and male fertility
Source: Sci Adv. 2023 Jan 25;9(4):eade7607. doi: 10.1126/sciadv.ade7607 (PMC9876558; doi:10.1126/sciadv.ade7607)
Supplement: Supplementary file 1 — Figs. S1 to S6 Tables S1 to S3 [file sciadv.ade7607_sm.pdf]

Science Advances
AAAS

Supplementary Materials for

Testis-enriched ferlin, FER1L5, is required for Ca^{2+}-activated acrosome reaction and male fertility

Akane Morohoshi *et al.*

Corresponding author: Haruhiko Miyata, hmiya003@biken.osaka-u.ac.jp;
Masahito Ikawa, ikawa@biken.osaka-u.ac.jp

Sci. Adv. **9**, eade7607 (2023)
DOI: 10.1126/sciadv.ade7607

This PDF file includes:

Figs. S1 to S6
Tables S1 to S3

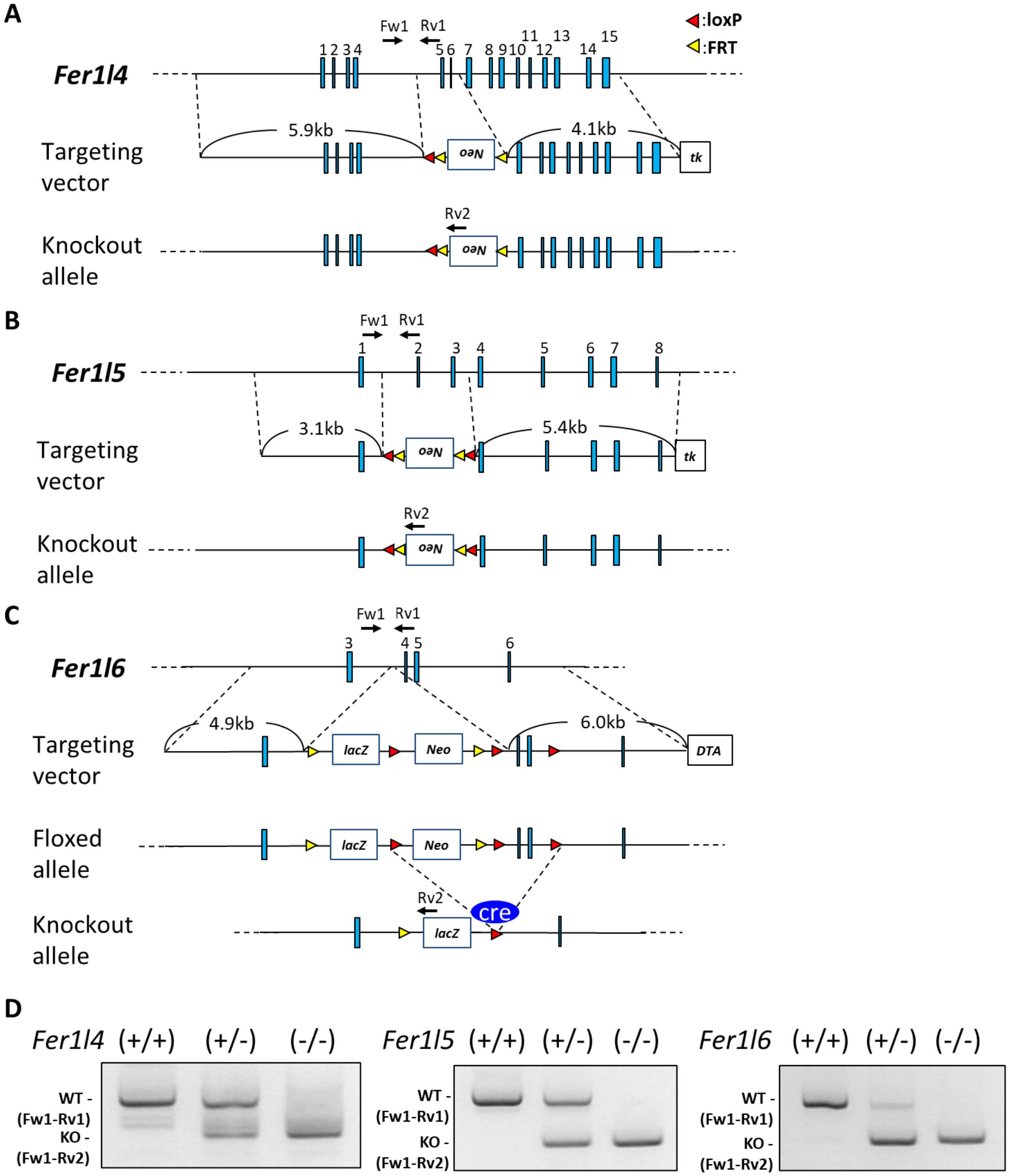

Fig. S1. Generation of *Fer1l4, Fer1l5,* and *Fer1l6* mutant mice.

(A) Targeting scheme for generating *Fer1l4* mutant mice. Exon 5 and 6 were replaced with a neomycin resistance cassette (Neo). **(B)** Targeting scheme for generating *Fer1l5* mutant mice. Exon 2 and 3 were replaced with Neo. **(C)** Targeting scheme for generating *Fer1l6* mutant mice. Exon 4 and 5 were flanked by loxP sites. Neo, exon 4 and 5 were removed in germ-line cells by mating floxed mice with *CAG-Cre* Tg mice. **(D)** Genotyping of *Fer1l4*, *Fer1l5*, and *Fer1l6* mice. Primers shown in fig. S1A-C were used.

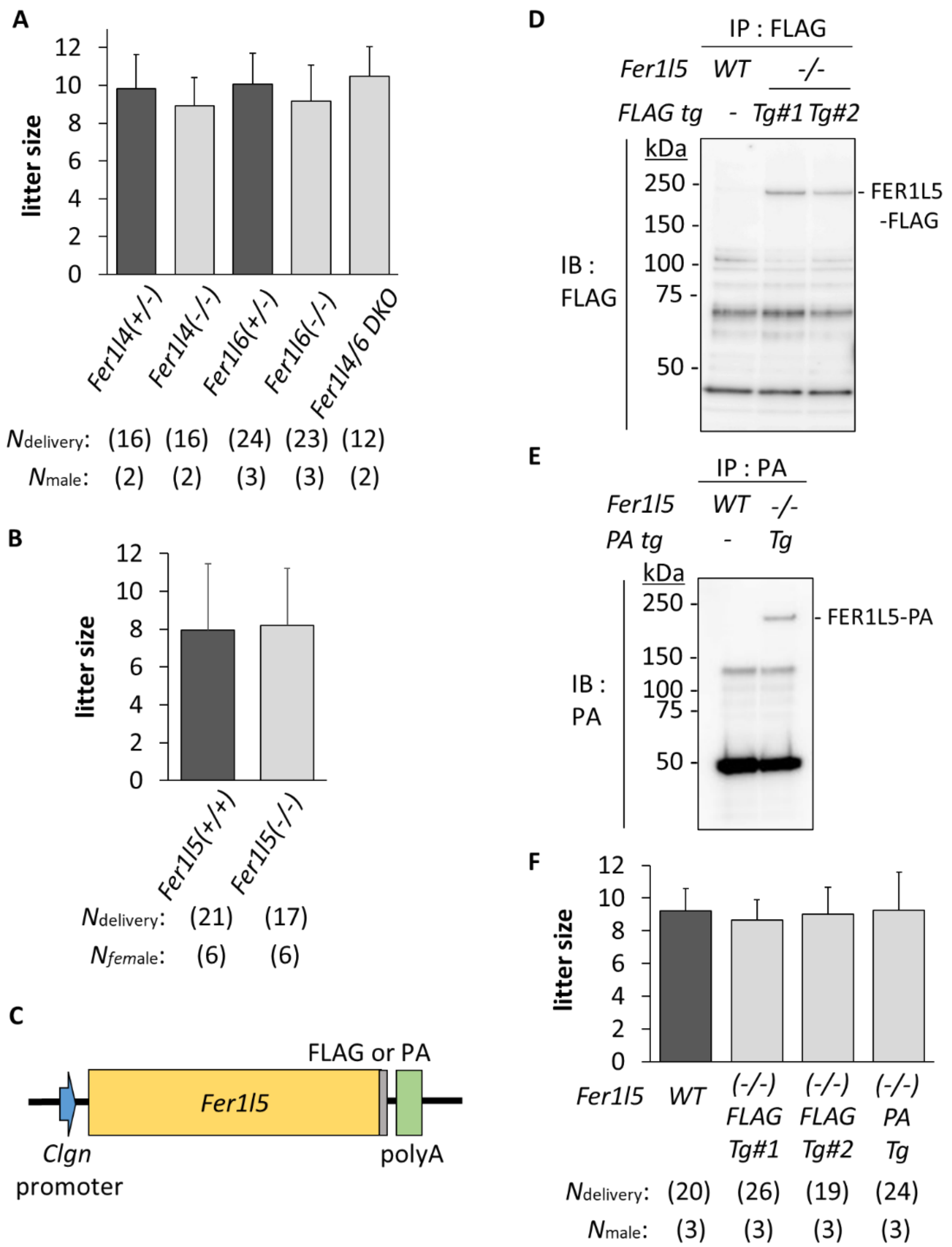

Fig. S2. Fertility of *Fer1l4, Fer1l6* mutant mice and *Fer1l5* transgenic mice.

(A) Fertility of *Fer1l4*, *Fer1l6*, and *Fer1l4/6* mutant male mice. These mutant mice were fertile. **(B)** Fertility of *Fer1l5* mutant female mice. *Fer1l5* mutant females were fertile. **(C)** The construct of transgene. *Fer1l5-FLAG* or *Fer1l5-PA* is expressed under the *Clgn* promoter. **(D)** FER1L5-FLAG was detected after immunoprecipitation with anti-FLAG antibody in *Fer1l5-FLAG* Tg mice (line #1 and #2). **(E)** FER1L5-PA was detected after immunoprecipitation with anti-PA antibody in *Fer1l5-PA* Tg mice. **(F)** Fertility of *Fer1l5-FLAG* and *Fer1l5-PA* transgenic mice. *Fer1l5-FLAG* and *Fer1l5-PA* transgenes rescued the fertility of *Fer1l5* mutant mice.

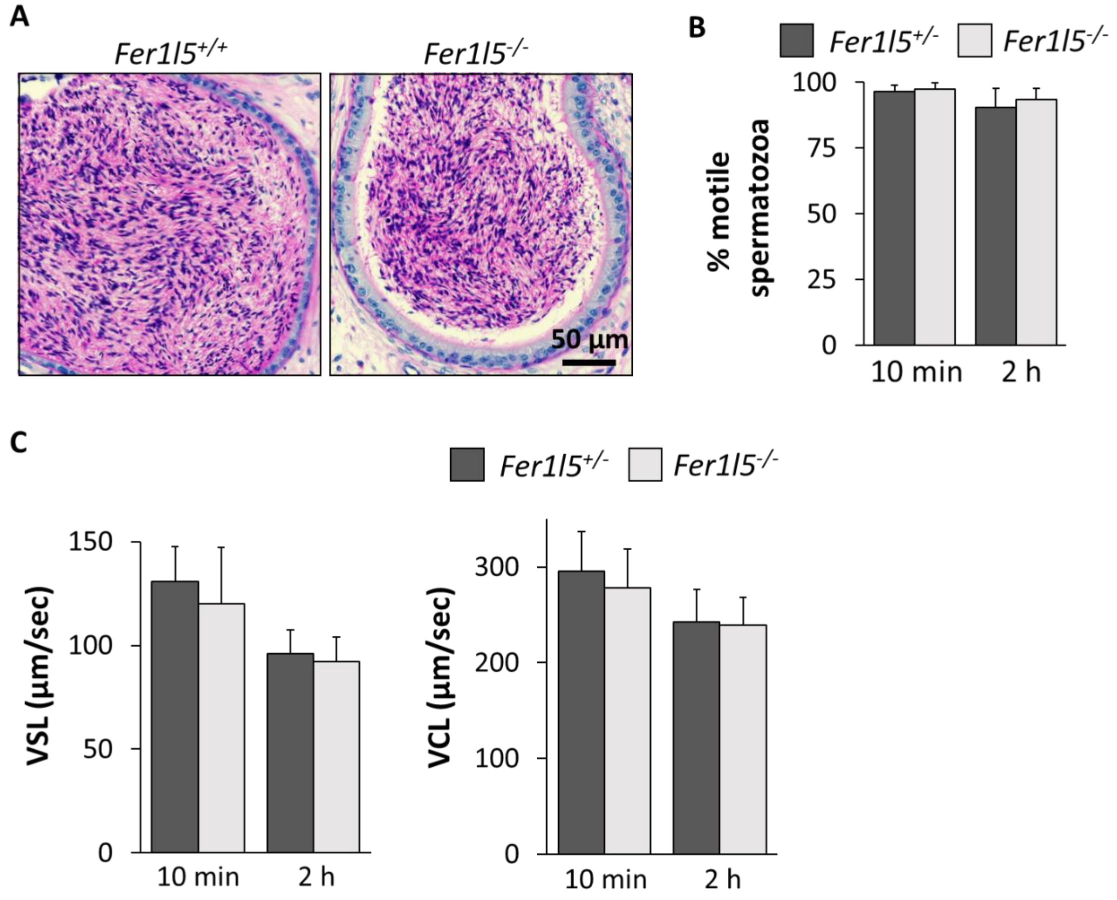

Fig. S3. Epididymal morphology and sperm motility.

(A) PAS staining of cauda epididymides. No overt abnormalities were found in *Fer1l5* mutant mice. **(B)** Percentages of motile spermatozoa. There were no significant differences between control and *Fer1l5* mutant mice. n = 5 males each for the control and *Fer1l5* mutant mice. **(C)** VSL (straight-line velocity) and VCL (curvilinear velocity) of sperm motility were analyzed. There were no significant differences in all the velocity parameters between control and *Fer1l5* mutant mice. n = 7 males for the control and n = 9 males for *Fer1l5* mutant mice.

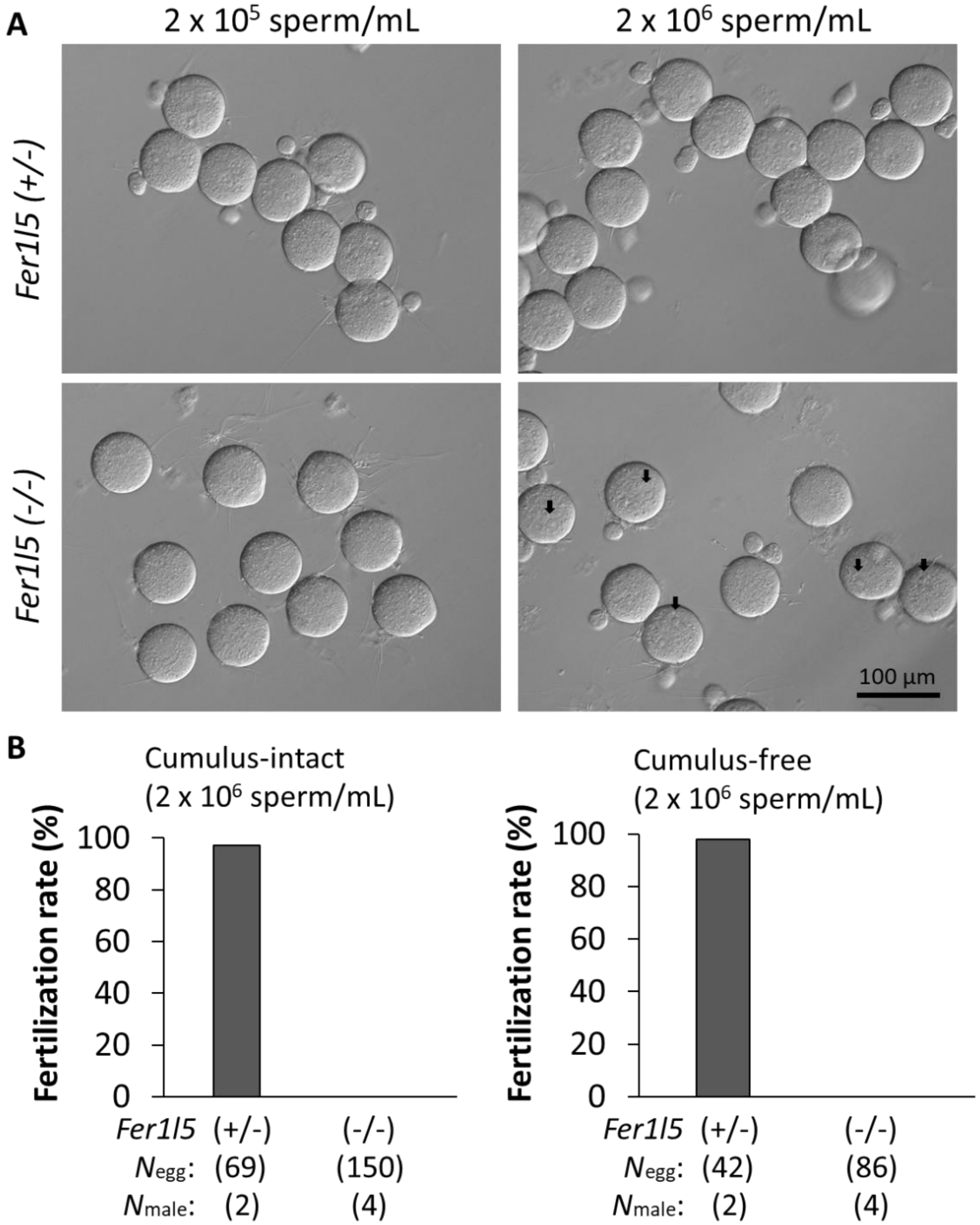

Fig. S4. *In vitro* fertility is impaired in *Fer1l5* mutant male mice.

(**A**) IVF with ZP-free oocytes. In *Fer1l5* mutant spermatozoa, no fertilization (pronuclei) was observed at a final density of 2×10^5 spermatozoa/mL, but pronuclei (arrows) were observed at a final density of 2×10^6 spermatozoa/mL. **(B)** IVF with cumulus-intact and cumulus-free oocytes. Ten times more spermatozoa than Fig. 3B and 3C were used for insemination.

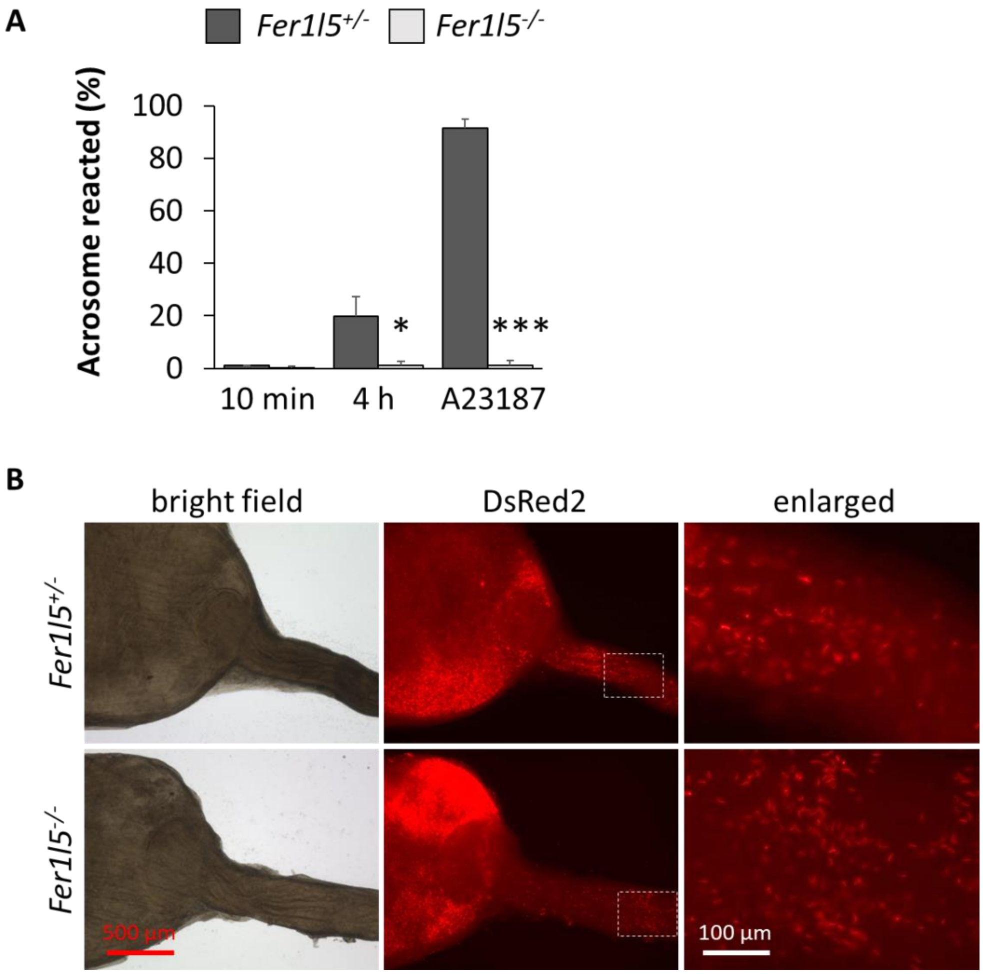

Fig. S5. Acrosome reaction and migration of *Fer1l5* mutant spermatozoa.

(A) The acrosome reaction rates were analyzed using RBGS mice after 10 minutes and 4 hours incubation in capacitation medium. After 4 hours incubation, Ca^{2+} ionophore A23187 was added to induce the acrosome reaction. n = 3 males for each genotype. *P < 0.05, and ***P < 0.001 (Student's t test). **(B)** Observation of spermatozoa at the junction between the uterus and oviduct 2 hours after observing vaginal plugs. Higher magnification images of the white boxed areas are shown to the right. *Fer1l5* mutant spermatozoa was observed in the oviduct.

A

Protein identification probability of FER1L5 = 74%

FER1L5 amino acid sequence	Peptide identification probability
NKTTSGVRGYLK	6%
SIRNPRDPALLQQWEK	10%
MTLEMLSER	78%

B

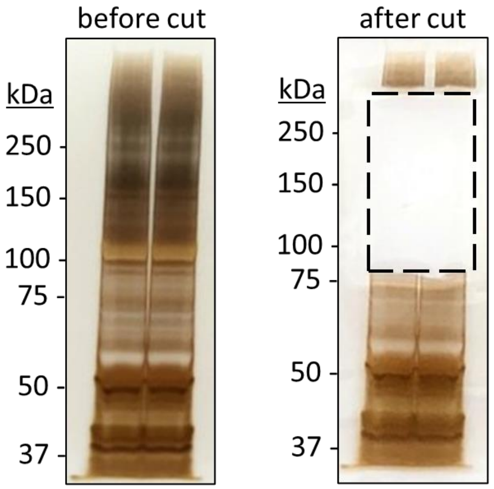

C

Protein identification probability of FER1L5 = 99%

FER1L5 amino acid sequence	Peptide identification probability
SQQDSVMKDLSQEVTQVEMQYYRQK	51%
SQLLQQLAQMAK	98%
SQLLQQLAQMAKEAKPVNMVGTAKEWLHR	6%
VKMTLEMLSER	21%

Fig. S6. Mass spectrometry analysis of proteins obtained from cauda epididymal spermatozoa.

(A) Sperm lysates were subjected to mass spectrometry analysis. **(B)** A gel fragment above 75 kDa was cut out for mass spectrometry analysis. **(C)** The gel fragment obtained in fig. S6B was subjected to mass spectrometry analysis.

Table S1. Primer and gRNA sequences used in this study.

Primers and gRNAs	Sequences
Fer1l4 RT-PCR Fw	TGGGCACTTCCACCAAAAGTG
Fer1l4 RT-PCR Rv	GGGTCAATCATGGTGGCCTC
Fer1l5 RT-PCR Fw	GCTGGCACAAAGACCGAAAG
Fer1l5 RT-PCR Rv	ACAGGAGTGGCTGACTTGTG
Fer1l6 RT-PCR Fw	AACATCGACCCGGTTGTGAC
Fer1l6 RT-PCR Rv	CTTTTGCACAGAGGTTCGCC
Actb RT-PCR Fw	AAGTGTGACGTTGACATCCG
Actb RT-PCR Rv	GATCCACATCTGCTGGAAGG
Fer1l4 long arm Fw	ATGCATTCTTGAAGCAGTAGTGCTCCTTTGT
Fer1l4 long arm Rv	CTCGAGGAAGGTGTTCTCTAACTTCTACACA
Fer1l4 short arm Fw	CTTAAGATAAGGTCAGAAGTCAACCCTTACACC
Fer1l4 short arm Rv	GGCGCGCCGAACTGACCTCCTTAATATATACC
Fer1l5 long arm Fw	TTAATTAAAGCGATGAGGATGGCCGAGA
Fer1l5 long arm Rv	GGTACCTCTGGGGTTCCGGGTGTTAGG
Fer1l5 short arm Fw	GCGGCCGCGGTGACAGCAGATACTTGCAAGG
Fer1l5 short arm Rv	GTCGACATGTGTCCTTCCTACTAGACGTGA
Fer1l4 genotyping Fw1	AAAGCAGCCAGTGCTCTTAACC
Fer1l4 genotyping Rv1	GAAGTACCAACATCTTGCACACC
Fer1l4 genotyping Rv2	CTTGACGAGTTCTTCTGAGG
Fer1l5 genotyping Fw1	TAGGGCATTCCCTCAATCTCTGCT
Fer1l5 genotyping Rv1	CATCATATCTGCAAAGACACCAAGG
Fer1l5 genotyping Rv2	CTTGACGAGTTCTTCTGAGG
Fer1l6 genotyping Fw1	CCATCATTGCCTCTGCCTTTCT
Fer1l6 genotyping Rv1	CCATCATGGATCTTAGTCAGCAGT
Fer1l6 genotyping Rv2	CACAACGGGTTCTTCTGTTAGTCC

Table S2. Antibodies used in this study.

Antibodies	Sources	ID#
Mouse anti-FLAG (M2)	Sigma-Aldrich	F1804
Mouse anti-Phosphotyrosine	Sigma-Aldrich	05-1050
Mouse anti-Tubulin	Sigma-Aldrich	T5168
Rabbit anti-ACTB	Medical & Biological Laboratories	PM053
Rabbit anti-FLAG (PM020)	Medical & Biological Laboratories	PM020
Rat anti-ADAM1B	Ikawa et al (50)	KS107-158
Rat anti-IZUMO1	Ikawa et al (50)	KS64–125
Rat anti-PA	FUJIFILM Wako Pure Chemical	012-25863
Anti-rat IgG Alexa 488	Thermo Fisher Scientific	A11006
Anti-mouse IgG HRP	Jackson ImmunoResearch	115-036-062
Anti-rabbit IgG HRP	Jackson ImmunoResearch	111-036-045
Anti-rat IgG HRP	Jackson ImmunoResearch	112-035-167

Table S3. Gene-manipulated mice used in this study.

Mouse lines	ID#
Fer1l4 mutant mice: C57BL/6N-Fer1l4<tm1Osb>/5A	RIKEN (RBRC06478), CARD (2487)
Fer1l5 mutant mice: STOCK-Fer1l5<tm1Osb>/75	RIKEN (RBRC09992), CARD (2547)
Fer1l6 mutant mice: STOCK Fer1l6<tm1a(KOMP)Osb>/80	RIKEN (RBRC05970), CARD (2179)
Fer1l5-FLAG transgenic mice #1: STOCK Tg(Clgn-Fer1l5/Flag)3Osb	RIKEN (RBRC10837), CARD (2817)
Fer1l5-FLAG transgenic mice #2: STOCK Tg(Clgn-Fer1l5/Flag)1Osb	RIKEN (RBRC10130), CARD (2603)
Fer1l5-PA transgenic mice: STOCK Tg(Clgn-Fer1l5/PA/1D4)1Osb	RIKEN (RBRC11653), CARD (3210)
CAG-Cre transgenic mice: B6.Cg-Tg(CAG-cre)CZ-MO2Osb	RIKEN (RBRC01828), CARD (1142)
RBGS mice: B6D2-Tg(CAG/Su9-DsRed2,Acr3-EGFP)RBGS002Osb	RIKEN (RBRC03743), CARD (1268)
